# Supplementary material for: A Survey-Based Study Investigating Opinions on Genetic Research Among Swedish Autistic Individuals and Parents of Autistic Children
Source: Autism. 2026 Jun 23;30(8):2064–78. doi: 10.1177/13623613261458410 (PMC13392166; doi:10.1177/13623613261458410)
Supplement: sj-docx-1-aut-10.1177_13623613261458410 – Supplemental material for A Survey-Based Study Investigating Opinions on Genetic Research Among Swedish Autistic Individuals and Parents of Autistic Children [file sj-docx-1-aut-10.1177_13623613261458410.docx]

# Supplementary Tables

### Supplementary Table 1. Survey questions on opinions on genetic research included in this study

| Survey for autistic adults | | | |
| --- | --- | --- | --- |
|  | Question | | Response options |
| 1 | I think research that aims to find genetic causes of ASD is positive. | | To a very large extent  To a large extent  Not that much  Not at all  Don’t know / prefer not to answer |
| 2 | I would participate in research aiming to find genetic causes of ASD. | | Yes  No  Don’t know / prefer not to answer |
| 3 | If I participate in genetic research on ASD, I would like to: | | Yes  No  Maybe  Don’t know / prefer not to answer |
|  | A. | Receive the results at the individual level (that is, the results of the analysis conducted specifically for you). |  |
|  | B. | Be informed if researchers find that I have an increased risk for any other disease or condition besides ASD. |  |
|  | C. | Only receive results at the group level (that is, not the results of the analysis conducted specifically for you). |  |
|  | D. | Contribute to new knowledge that may hopefully help other children, adolescents, and adults with ASD in the future. |  |
| 4 | I believe that research on ASD will lead to ASD being preventable in the future, or that symptoms of ASD can be treated through new medications or other interventions. | | To a very large extent  To a large extent  Not that much  Not at all  Don’t know / prefer not to answer |
| 5 | I am worried that the results from research identifying genetic variants that increase the likelihood of ASD could be misused by others.  If yes, please describe what you think would be a misuse in the field below. | | To a very large extent  To a large extent  Not that much  Not at all  Don’t know / prefer not to answer |
| Survey for parents to autistics | | | |
| 1 | I think research that aims to find genetic causes of ASD is positive. | | To a very large extent  To a large extent  Not that much  Not at all  Don’t know / prefer not to answer |
| 2 | I would allow my child to participate in research aiming to find genetic causes of ASD. | | Yes  No  Don’t know / prefer not to answer |
| 3 | If my child were to participate in genetic research on ASD, it is important that: | | Yes  No  Maybe  Don’t know / prefer not to answer |
|  | A. | We receive the results at the individual level (that is, the results of the analysis conducted specifically for your child). |  |
|  | B. | We are informed if it is found that the child has an increased risk for another disease or condition besides ASD. |  |
|  | C. | We receive the results only at the group level (that is, not the results of the analysis conducted specifically for your child). |  |
|  | D. | The research leads to new knowledge that may hopefully help other children with ASD in the future. |  |
| 4 | I believe that current genetic research (mapping of risk genes/variants for ASD) may lead to a future where fetuses showing a high likelihood of developing ASD could be selected against through prenatal diagnostics. | | To a very large extent  To a large extent  Not that much  Not at all  Don’t know / prefer not to answer |
| 5 | I believe that research on ASD will lead to ASD being preventable in the future, or that symptoms of ASD can be treated through new medications or other interventions. | | To a very large extent  To a large extent  Not that much  Not at all  Don’t know / prefer not to answer |

### Supplementary Table 2**.** Demographic differences between respondents who left a comment and those who did not in the autism-group.

| **Characteristics** | Comment with a fear regarding genetic research | | P-value* |
| --- | --- | --- | --- |
|  | Yes (n=103)  Number (%) | No (n=109)  Number (%) |  |
| **Gender** | | | |
| Woman | 73 (70.9) | 86 (78.9) | 0.23 |
| Man | 21 (20.4) | 21 (19.3) | 0.97 |
| Non-binary | 6 (5.8) | <5 | 0.06 |
| Unsure | <5 | <5 | 1 |
| Prefer not to say | <5 | 0 | 0.98 |
|  |  |  |  |
| **Age** | | | |
| 15 – 18 | <5 | 7 (6.4) | 0.43 |
| 19 – 25 | 12 (11.7) | 15 (13.8) | 0.92 |
| 26 – 35 | 33 (32.0) | 36 (33.0) | 1 |
| 36 – 45 | 25 (24.3) | 27 (24.8) | 1 |
| 46 – 55 | 20 (19.4) | 12 (11.0) | 0.09 |
| 56 – 65 | 8 (7.8) | 11 (10.1) | 0.82 |
| > 65 | <5 | <5 | 0.23 |
|  |  |  |  |
| **Income** | | | |
| 0 – 19 999 | 30 (29.1) | 46 (42.2) | 0.07 |
| 20 000 – 39 999 | 28 (27.2) | 32 (29.4) | 0.84 |
| 40 000 – 59 999 | 18 (17.5) | 13 (11.9) | 0.34 |
| 60 000 – 89 999 | 16 (15.5) | 11 (10.1) | 0.33 |
| > 90 000 | <5 | <5 | 0.63 |
| Prefer not to answer | 7 (6.8) | 5 (4.6) | 0.69 |
|  |  |  |  |
| **Education** | | | |
| Primary school | 10 (9.7) | \| 30 (27.5) \| \| --- \| | 0.0017 |
| Secondary school | 21 (20.4) | 31 (28.4) | 0.23 |
| Post-secondary school | 9 (8.7) | 7 (6.4) | 0.71 |
| University studies | 20 (19.4) | 13 (11.9) | 0.19 |
| University degree | 42 (40.8) | 27 (24.8) | 0.019 |
| Prefer not to say | <5 | <5 | 1 |
|  |  |  |  |
| **Comorbidity with other neurodevelopmental disorders** | | | |
| ADHD | 30 (29.1) | 34 (31.2) | 0.72 |
| ADD | 21 (20.4) | 17 (15.6) | 0.56 |
| Intellectual disability | <5 | <5 | 0.63 |
| Tourettes syndrome | <5 | <5 | 1 |
| Dyslexia | 9 (8.7) | 10 (9.2) | 1 |
| Dyscalculia | 5 (4.9) | <5 | 0.21 |
| No | 47 (45.6) | 50 (45.9) | 0.88 |
| Prefer not to answer | <5 | <5 | 1 |
|  |  |  |  |
| **Comorbidity with other psychiatric disorders** | | | |
| Depression | 62 (60.2) | 65 (59.6) | 0.95 |
| Anxiety | 58 (56.3) | 65 (59.6) | 0.88 |
| Specific phobia | 10 (9.7) | 9 (8.3) | 0.87 |
| Social phobia | 16 (15.5) | 28 (25.7) | 0.14 |
| Sleeping problems | 51 (49.5) | 47 (43.1) | 0.47 |
| Exhaustion syndrome | 34 (33) | 36 (33.0) | 1 |
| OCD | 13 (12.6) | 13 (11.9) | 1 |
| Anorexia | <5 | 8 (7.3) | 0.27 |
| Bipolar disorder | <5 | 6 (5.5) | 0.34 |
| Psychosis | 0 | <5 | 0.51 |
| Schizophrenia | 0 | <5 | 1 |
| PTSD | 20 (19.4) | 14 (12.8) | 0.27 |
| Alcohol abuse | 0 | <5 | 0.15 |
| Drug abuse | <5 | <5 | 0.67 |
| Other | 18 (17.5) | 5 (4.6) | **0.0063** |
| No | 12 (11.7) | 16 (14.7) | 0.70 |
| Prefer not to answer | <5 | <5 | 1 |

*

### Supplementary Table 3. Association between education and income with concerns regarding genetic research in autism.

| Variable | Estimate | Std. Error | t-value | p-value |
| --- | --- | --- | --- | --- |
| Autism-group | | | | |
| Income | -0.170 | 0.0656 | -2.59 | **0.011** |
| Education | -0.177 | 0.0491 | -3.60 | **0.0004** |
| Parent-group | | | | |
| Income | 0.0171 | 0.0281 | 0.610 | 0.54 |
| Education | -0.0230 | 0.0232 | -0.991 | 0.67 |

### Supplementary File 1. Analysis codes used for Figure 1.

1. library(tidyverse)

2. library(readxl)

3. library(cowplot)

4.

5. # ---------------------------

6. # Load data

7. # ---------------------------

8. parents_data <- read_excel("…/dataparents.xlsx", sheet = "Data")

9. adults_data <- read_excel("…/dataautisticadults.xlsx", sheet = "Data")

10.

11. # ---------------------------

12. # Questions & labels

13. # ---------------------------

14. questions_adults <- c("VAR26_1_", "VAR26_2_", "VAR26_3_", "VAR26_4_")

15. questions_parents <- c("VAR37_1_", "VAR37_2_", "VAR37_3_", "VAR37_4_")

16.

17. statement_labels <- c(

18. "Receive results at\nindividual level",

19. "Be informed of\nother risks",

20. "Receive results at\ngroup level",

21. "Contribute to\nnew knowledge"

22. )

23.

24. answer_labels <- c("Yes", "No", "Maybe", "Don’t know /\nprefer not\nto answer")

25.

26. # ---------------------------

27. # Reshape function

28. # ---------------------------

29. reshape_checkbox_matrix <- function(df, question_prefixes, statements) {

30. df <- df %>% mutate(rowid = row_number())

31. map_dfr(seq_along(question_prefixes), function(i) {

32. q_prefix <- question_prefixes[i]

33. label <- statements[i]

34. cols <- paste0(q_prefix, 1:4)

35. df %>%

36. select(rowid, all_of(cols)) %>%

37. pivot_longer(cols = -rowid, names_to = "col", values_to = "val") %>%

38. filter(val == 1) %>%

39. mutate(

40. statement = label,

41. response = case_when(

42. grepl("_1$", col) ~ "Yes",

43. grepl("_2$", col) ~ "No",

44. grepl("_3$", col) ~ "Maybe",

45. grepl("_4$", col) ~ "Don’t know /\nprefer not\nto answer"

46. ),

47. response = factor(response, levels = answer_labels)

48. ) %>%

49. select(rowid, statement, response)

50. })

51. }

52.

53. # ---------------------------

54. # Build data

55. # ---------------------------

56. adults_long <- reshape_checkbox_matrix(adults_data, questions_adults, statement_labels) %>%

57. mutate(group = "Autism-group")

58.

59. parents_long <- reshape_checkbox_matrix(parents_data, questions_parents, statement_labels) %>%

60. mutate(group = "Parent-group")

61.

62. all_data <- bind_rows(adults_long, parents_long)

63.

64. # ---------------------------

65. # Percent table

66. # ---------------------------

67. plot_data <- all_data %>%

68. group_by(statement, group, response) %>%

69. summarise(n = n(), .groups = "drop") %>%

70. group_by(statement, group) %>%

71. mutate(percent = n / sum(n) * 100) %>%

72. ungroup()

73.

74. # ---------------------------

75. # p-values per statement × response

76. # ---------------------------

77. compute_pairwise_pvals <- function(adults_long, parents_long, statements, responses) {

78. res_list <- vector("list", length(statements))

79. names(res_list) <- statements

80. for (stmt in statements) {

81. totals_ad <- sum(adults_long$statement == stmt)

82. totals_pa <- sum(parents_long$statement == stmt)

83. pv <- numeric(length(responses))

84. names(pv) <- responses

85. for (resp in responses) {

86. ad_yes <- sum(adults_long$statement == stmt & adults_long$response == resp)

87. pa_yes <- sum(parents_long$statement == stmt & parents_long$response == resp)

88. tab <- matrix(c(ad_yes, totals_ad - ad_yes,

89. pa_yes, totals_pa - pa_yes),

90. nrow = 2, byrow = TRUE)

91. use_fisher <- any(tab < 5)

92. test <- if (use_fisher) fisher.test(tab) else chisq.test(tab)

93. pv[resp] <- test$p.value

94. }

95. res_list[[stmt]] <- pv

96. }

97. return(res_list)

98. }

99.

100. pvals_list <- compute_pairwise_pvals(adults_long, parents_long, statement_labels, answer_labels)

101.

102. format_p <- function(p) {

103. if (p < 0.001) "p < 0.001" else paste0("p = ", formatC(p, digits = 3, format = "f"))

104. }

105. p_to_stars <- function(p) {

106. if (p < 0.001) "***"

107. else if (p < 0.01) "**"

108. else if (p < 0.05) "*"

109. else ""

110. }

111.

112. # ---------------------------

113. # Colors

114. # ---------------------------

115. # NOTE: The group name used here must match the new name above

116. group_colors <- c("Autism-group" = "skyblue", "Parent-group" = "orange")

117.

118. # ---------------------------

119. # Plot function

120. # ---------------------------

121. plot_statement <- function(stmt, title_label, show_legend = FALSE, inside_threshold = 8) {

122.

123. df <- plot_data %>% filter(statement == stmt)

124. df$response <- factor(df$response, levels = answer_labels)

125. df$group <- factor(df$group, levels = c("Autism-group", "Parent-group"))

126.

127. # Compute for each response the max bar height (for p-value placement)

128. max_per_response <- df %>%

129. group_by(response) %>%

130. summarise(max_pct = max(percent, na.rm = TRUE), .groups = "drop")

131.

132. # Build pvalue display df

133. pvals_resp <- pvals_list[[stmt]]

134. pval_df <- tibble(

135. response = factor(names(pvals_resp), levels = answer_labels),

136. p = as.numeric(pvals_resp),

137. p_lab = sapply(as.numeric(pvals_resp), format_p),

138. stars = sapply(as.numeric(pvals_resp), p_to_stars)

139. ) %>%

140. left_join(max_per_response, by = "response") %>%

141. # Customized Y-position logic based on graph (stmt) and response

142. mutate(y = case_when(

143. # Custom low offset for Graph C and "No"

144. stmt == "Receive results at\ngroup level" & response == "No" ~ max_pct + 8.0,

145.

146. # Custom low offset for Graph B and C - "Maybe"

147. (stmt == "Be informed of\nother risks" | stmt == "Receive results at\ngroup level") & response == "Maybe" ~ max_pct + 8.5,

148.

149. # Default High offset (14) for all other low-percent responses

150. response != "Yes" ~ max_pct + 14,

151.

152. # Standard offset (9) for 'Yes' responses

153. TRUE ~ max_pct + 9.0

154. ))

155.

156. # Decide percent label placement: inside or outside

157. df <- df %>%

158. mutate(

159. label_text = paste0(round(percent, 1), "%"),

160. inside = percent >= inside_threshold,

161. label_y = ifelse(inside, percent/2, percent + 1.8),

162. label_color = ifelse(inside, "black", "black"),

163. label_vjust = ifelse(inside, 0.5, 0)

164. )

165.

166. # Basic plot (grouped bars)

167. p <- ggplot(df, aes(x = response, y = percent, fill = group)) +

168. geom_col(position = position_dodge(width = 0.8), width = 0.7) +

169.

170. # Percent labels

171. geom_text(

172. data = df,

173. aes(x = response, y = label_y, label = label_text, group = group),

174. position = position_dodge(width = 0.8),

175. color = df$label_color,

176. size = 3.6,

177. vjust = df$label_vjust,

178. inherit.aes = FALSE

179. ) +

180.

181. # p-values above bars

182. geom_text(

183. data = pval_df,

184. aes(x = response, y = y, label = paste0(p_lab, " ", stars)),

185. inherit.aes = FALSE,

186. size = 3.6,

187. fontface = "plain" # Ensure p-values are not bold

188. ) +

189.

190. scale_fill_manual(values = group_colors) +

191. scale_y_continuous(expand = c(0, 0),

192. limits = c(0, 105)) + # Y-axis limit fixed to 105

193. labs(title = title_label, x = NULL, y = "Percentage", fill = NULL) +

194.

195. theme_minimal(base_size = 14) +

196. theme(

197. plot.title = element_text(size = 15, face = "bold", hjust = 0.5),

198. legend.position = if (show_legend) "bottom" else "none",

199. axis.text.x = element_text(size = 11),

200. axis.text.y = element_text(size = 11)

201. )

202.

203. return(p)

204. }

205. # ---------------------------

206. # Final 4 plots

207. # ---------------------------

208. p1 <- plot_statement(statement_labels[1], "Receive results at individual level")

209. p2 <- plot_statement(statement_labels[2], "Be informed of other risks")

210. p3 <- plot_statement(statement_labels[3], "Receive results at group level")

211. p4 <- plot_statement(statement_labels[4], "Contribute to new knowledge")

212.

213. legend_temp <- plot_statement(statement_labels[1], "legend", show_legend = TRUE)

214. legend <- get_legend(legend_temp)

215.

216. combined <- plot_grid(

217. p1, p2, p3, p4,

218. ncol = 2,

219. labels = c("A", "B", "C", "D"),

220. label_size = 18,

221. label_fontface = "bold",

222. label_x = 0.02,

223. label_y = 0.98

224. )

225.

226. final_plot <- plot_grid(combined, legend, ncol = 1, rel_heights = c(1, 0.08))

227.

228. print(final_plot)

229.

230. # -------------------------------------------------

231. # PRINT SUMMARY P-VALUE TABLE FOR LOGBOOK

232. # -------------------------------------------------

233.

234. pval_table <- map_dfr(names(pvals_list), function(stmt) {

235. tibble(

236. Statement = stmt, a

237. Response = names(pvals_list[[stmt]]),

238. P_value = as.numeric(pvals_list[[stmt]]),

239. P_formatted = sapply(as.numeric(pvals_list[[stmt]]), function(p) {

240. if (p < 0.001) "p < 0.001" else paste0("p = ", formatC(p, digits = 4, format = "f"))

241. })

242. )

243. })

244.

245. cat("\n==================== P-VALUE SUMMARY ====================\n")

246. print(pval_table, n = Inf)

247. cat("==========================================================\n")

248.

### Supplementary File 2. Analysis codes used for Figure 2.

1. # Load necessary libraries

2. library(readxl)

3. library(dplyr)

4. library(ggplot2)

5. library(cowplot)

6.

7. # --- Global collector for summary table ---

8. pval_summary_list <- list()

9.

10. # --- Load data ---

11. parents_data <- read_excel("…/dataparents.xlsx", sheet = "Data")

12. adults_data <- read_excel("…/dataautisticadults.xlsx", sheet = "Data")

13.

14. # --- Helper functions ---

15. preprocess_data <- function(data, column, group_labels, levels_order) {

16. data %>%

17. filter(!!sym(column) %in% names(group_labels)) %>%

18. mutate(Group = factor(group_labels[as.character(!!sym(column))], levels = levels_order)) %>%

19. group_by(Group) %>%

20. summarise(n = n(), .groups = "drop") %>%

21. mutate(Percentage = n / sum(n) * 100)

22. }

23.

24. calculate_pvals <- function(raw_parents, raw_adults, var_parent, var_adult, group_labels) {

25. pvals <- c()

26. for (grp_label in unique(group_labels)) {

27. if (is.na(grp_label) | grp_label == "") next

28. keys <- names(group_labels[group_labels == grp_label])

29. parents_yes <- sum(raw_parents[[var_parent]] %in% keys, na.rm = TRUE)

30. parents_no <- sum(!(raw_parents[[var_parent]] %in% keys), na.rm = TRUE)

31. adults_yes <- sum(raw_adults[[var_adult]] %in% keys, na.rm = TRUE)

32. adults_no <- sum(!(raw_adults[[var_adult]] %in% keys), na.rm = TRUE)

33. tbl <- matrix(c(parents_yes, parents_no, adults_yes, adults_no), nrow = 2)

34. if (any(tbl < 5)) test <- fisher.test(tbl) else test <- chisq.test(tbl)

35. pvals[grp_label] <- test$p.value

36. }

37. return(pvals)

38. }

39.

40. # --- Graph function (with p-value storage + printing) ---

41. create_bar_graph <- function(data_parents, data_adults, title,

42. raw_parents, raw_adults, var_parent, var_adult, group_labels,

43. show_legend = FALSE) {

44.

45. data_parents$GroupType <- "Parent-group"

46. data_adults$GroupType <- "Autism-group"

47. data_combined <- bind_rows(data_parents, data_adults)

48.

49. # --- P-values ---

50. pvals <- calculate_pvals(raw_parents, raw_adults, var_parent, var_adult, group_labels)

51.

52. # Store in global summary table

53. pval_summary_list[[title]] <<- pvals

54.

55. # Print p-values immediately

56. cat("\n--- P-values for:", title, "---\n")

57. print(pvals)

58.

59. # Stars for annotations

60. stars <- sapply(pvals, function(p) {

61. if (is.na(p)) return("")

62. else if (p < 0.001) return("***")

63. else if (p < 0.01) return("**")

64. else if (p < 0.05) return("*")

65. else return("")

66. })

67.

68. # P-value annotation placement

69. pval_df <- data_combined %>%

70. group_by(Group) %>%

71. summarise(y = max(Percentage) + ifelse(Group %in% c("Not at all", "Don’t know /\nprefer not\nto answer"), 10, 3),

72. .groups = 'drop') %>%

73. mutate(p_label = paste0("p=", formatC(pvals[Group], format = "f", digits = 3), stars[Group]))

74.

75. # Percentage annotations

76. perc_df <- data_combined %>%

77. mutate(

78. perc_label = paste0(round(Percentage, 1), "%"),

79. inside = Percentage >= 8,

80. y_perc = ifelse(inside, Percentage/2, Percentage + 1.8),

81. vjust_perc = ifelse(inside, 0.5, 0),

82. color_perc = "black"

83. )

84.

85. ggplot(data_combined, aes(x = Group, y = Percentage, fill = GroupType)) +

86. geom_bar(stat = "identity", position = position_dodge(width = 0.9)) +

87. geom_text(data = pval_df, aes(x = Group, y = y, label = p_label),

88. vjust = 0, size = 4, inherit.aes = FALSE) +

89. geom_text(data = perc_df,

90. aes(x = Group, y = y_perc, label = perc_label, group = GroupType,

91. vjust = vjust_perc, color = color_perc),

92. position = position_dodge(width = 0.9),

93. size = 4, inherit.aes = FALSE, show.legend = FALSE) +

94. scale_y_continuous(limits = c(0, 80), breaks = seq(0, 80, 20)) +

95. scale_fill_manual(values = c("Parent-group" = "orange", "Autism-group" = "skyblue")) +

96. scale_color_identity() +

97. labs(title = title, x = "", y = "Percentage") +

98. theme_minimal(base_size = 16) +

99. theme(

100. legend.position = if (show_legend) "bottom" else "none",

101. legend.title = element_blank(),

102. legend.text = element_text(size = 18),

103. plot.title = element_text(size = 18, face = "bold", hjust = 0.5),

104. axis.title.y = element_text(size = 16),

105. axis.text.x = element_text(size = 14, vjust = 0.5, hjust = 0.5),

106. axis.text.y = element_text(size = 14)

107. )

108. }

109.

110. # --- Define labels ---

111. group_labels_1_3_separate <- c(

112. "1" = "Very large", "2" = "Large",

113. "3" = "Not that much", "4" = "Not at all",

114. "5" = "Don’t know /\nprefer not\nto answer",

115. "6" = "Prefer not to answer"

116. )

117. levels_order_1_3_separate <- c("Very large", "Large", "Not that much", "Not at all", "Don’t know /\nprefer not\nto answer", "Prefer not to answer")

118.

119. group_labels_4 <- c("1" = "Yes", "2" = "No", "0" = "Don’t know /\nprefer not\nto answer")

120. levels_order_4 <- c("Yes", "No", "Don’t know /\nprefer not\nto answer")

121.

122. # --- Preprocess datasets ---

123. data1_parents_sep <- preprocess_data(parents_data, "VAR35", group_labels_1_3_separate, levels_order_1_3_separate)

124. data1_adults_sep <- preprocess_data(adults_data, "VAR24", group_labels_1_3_separate, levels_order_1_3_separate)

125. data2_parents_sep <- preprocess_data(parents_data, "VAR39", group_labels_1_3_separate, levels_order_1_3_separate)

126. data2_adults_sep <- preprocess_data(adults_data, "VAR27", group_labels_1_3_separate, levels_order_1_3_separate)

127. data3_parents_sep <- preprocess_data(parents_data, "VAR38", group_labels_1_3_separate, levels_order_1_3_separate)

128. data3_adults_sep <- preprocess_data(adults_data, "VAR28", group_labels_1_3_separate, levels_order_1_3_separate)

129. data4_parents_sep <- preprocess_data(parents_data, "VAR36", group_labels_4, levels_order_4)

130. data4_adults_sep <- preprocess_data(adults_data, "VAR25", group_labels_4, levels_order_4)

131.

132. # --- Create graphs ---

133. graph1a <- create_bar_graph(data1_parents_sep, data1_adults_sep,

134. "I think research that aims to find\n genetic causes of autism is positive",

135. parents_data, adults_data, "VAR35", "VAR24", group_labels_1_3_separate)

136.

137. graph2a <- create_bar_graph(data2_parents_sep, data2_adults_sep,

138. "I believe research will make autism \npreventable or treatable in the future",

139. parents_data, adults_data, "VAR39", "VAR27", group_labels_1_3_separate)

140.

141. graph3a <- create_bar_graph(data3_parents_sep, data3_adults_sep,

142. "I am worried that genetic research in autism could\n be misused by others/may lead to selection against\nfetuses with high likelihood of autism",

143. parents_data, adults_data, "VAR38", "VAR28", group_labels_1_3_separate)

144.

145. graph4 <- create_bar_graph(data4_parents_sep, data4_adults_sep,

146. "I/My child would participate in research aiming\n to find genetic causes of autism",

147. parents_data, adults_data, "VAR36", "VAR25", group_labels_4)

148.

149. # --- Extract legend ---

150. legend_plot <- create_bar_graph(data4_parents_sep, data4_adults_sep,

151. "Opinion on participating in genetic research",

152. parents_data, adults_data, "VAR36", "VAR25", group_labels_4, show_legend = TRUE)

153. legend <- get_legend(legend_plot)

154.

155. # --- Combine four plots ---

156. combined_plots <- plot_grid(

157. graph1a, graph2a, graph3a, graph4,

158. ncol = 2, align = "hv",

159. labels = c("A", "B", "C", "D"),

160. label_size = 18,

161. label_fontface = "bold",

162. label_x = 0.02,

163. label_y = 0.98

164. )

165.

166. # --- Add legend ---

167. final_plot <- plot_grid(combined_plots, legend, ncol = 1, rel_heights = c(1, 0.08))

168.

169. print(final_plot)

170.

171. # --- FINAL SUMMARY TABLE ---

172. cat("\n\n---------------- P-VALUE SUMMARY TABLE ----------------\n")

173.

174. summary_df <- do.call(rbind, lapply(names(pval_summary_list), function(name) {

175. row <- pval_summary_list[[name]]

176. data.frame(

177. Question = name,

178. t(as.data.frame(row)),

179. check.names = FALSE

180. )

181. }))

182.

183. print(summary_df, row.names = FALSE)

184.

185. cat("---------------------------------------------------------\n")

### Supplementary File 3. Analysis codes used for Supplementary Table 3.

1. # Load required libraries

2. library(readxl)

3. library(dplyr)

4. library(broom)

5. library(ggplot2)

6. library(gridExtra)

7.

8. # Define function to perform regression and tidy the results

9. perform_regression <- function(data, dependent_variable, independent_variable, dependent_label) {

10. model <- lm(data[[dependent_variable]] ~ data[[independent_variable]], data = data)

11. tidy_results <- tidy(model)

12. colnames(tidy_results)[1] <- "Variable"

13. colnames(tidy_results)[-1] <- c("Coefficient", "Std. Error", "t-score", "p-value")

14. tidy_results$Dependent_Variable <- dependent_label

15. return(tidy_results)

16. }

17.

18. # Load and filter data for adolescent/adults group

19. data_filtered_adults <- read_excel("…/dataautisticadults.xlsx", sheet = "Data") %>%

20. filter(VAR12 %in% 1:5, VAR13 %in% 1:5, VAR06 %in% 1:5, VAR28 %in% 1:4)

21.

22. # Perform regression analysis for adolescent/adults group

23. regression_results_adults <- bind_rows(

24. perform_regression(data_filtered_adults, "VAR12", "VAR28", "Income Adults/Adolescents"),

25. perform_regression(data_filtered_adults, "VAR13", "VAR28", "Education Adults/Adolescents"),

26. perform_regression(data_filtered_adults, "VAR06", "VAR28", "Stress Adults/Adolescents")

27. )

28.

29. # Plot the results for adolescent/adults group

30. print(

31. ggplot(regression_results_adults, aes(x = Coefficient, y = Dependent_Variable)) +

32. geom_point() +

33. geom_errorbarh(aes(xmin = Coefficient - `Std. Error`, xmax = Coefficient + `Std. Error`)) +

34. labs(x = "Coefficient", y = "Dependent Variable", title = "Regression Results for Adolescent/Adults Group")

35. )

36.

37. # Load and filter data for parents group

38. data_filtered_parents <- read_excel("…/dataparents.xlsx", sheet = "Data") %>%

39. filter(VAR12 %in% 1:5, VAR13 %in% 1:5, VAR20 %in% 1:5, VAR38 %in% 1:4)

40.

41. # Perform regression analysis for parents group

42. regression_results_parents <- bind_rows(

43. perform_regression(data_filtered_parents, "VAR12", "VAR38", "Income Parents"),

44. perform_regression(data_filtered_parents, "VAR13", "VAR38", "Education Parents"),

45. perform_regression(data_filtered_parents, "VAR20", "VAR38", "Stress Parents")

46. )

47.

48. # Plot the results for parents group

49. print(

50. ggplot(regression_results_parents, aes(x = Coefficient, y = Dependent_Variable)) +

51. geom_point() +

52. geom_errorbarh(aes(xmin = Coefficient - `Std. Error`, xmax = Coefficient + `Std. Error`)) +

53. labs(x = "Coefficient", y = "Dependent Variable", title = "Regression Results for Parents Group")

54. )

55.

56. # Combine regression results from both groups

57. combined_results <- bind_rows(regression_results_adults, regression_results_parents)

58.

59. # Print combined results

60. print(combined_results)

61.

62. # Plot combined results

63. print(

64. ggplot(combined_results, aes(x = Coefficient, y = Dependent_Variable)) +

65. geom_point() +

66. geom_errorbarh(aes(xmin = Coefficient - `Std. Error`, xmax = Coefficient + `Std. Error`)) +

67. facet_wrap(~Dependent_Variable, scales = "free_y") +

68. labs(x = "Coefficient", y = "Dependent Variable", title = "Combined Regression Results")

69. )

70.
